# Peptide Epimerization Machineries Found in Microorganisms

**DOI:** 10.3389/fmicb.2018.00156

**Published:** 2018-02-06

**Authors:** Yasushi Ogasawara, Tohru Dairi

**Affiliations:** Graduate School of Engineering, Hokkaido University, Sapporo, Japan

**Keywords:** biosynthesis, D-amino acid, epimerase, microorganism, natural products, peptide, peptidoglycan

## Abstract

D-Amino acid residues have been identified in peptides from a variety of eukaryotes and prokaryotes. In microorganisms, UDP-*N*-acetylmuramic acid pentapeptide (UDP-MurNAc-L-Ala-D-Glu-meso-diaminopimelate-D-Ala-D-Ala), a unit of peptidoglycan, is a representative. During its biosynthesis, D-Ala and D-Glu are generally supplied by racemases from the corresponding isomers. However, we recently identified a unique unidirectional L-Glu epimerase catalyzing the epimerization of the terminal L-Glu of UDP-MurNAc-L-Ala-L-Glu. Several such enzymes, introducing D-amino acid resides into peptides via epimerization, have been reported to date. This includes a L-Ala-D/L-Glu epimerase, which is possibly used during peptidoglycan degradation. In bacterial primary metabolisms, to the best of our knowledge, these two machineries are the only examples of peptide epimerization. However, a variety of peptides containing D-amino acid residues have been isolated from microorganisms as secondary metabolites. Their biosynthetic mechanisms have been studied and three different peptide epimerization machineries have been reported. The first is non-ribosomal peptide synthetase (NRPS). Excellent studies with dissected modules of gramicidin synthetase and tyrocidine synthetase revealed the reactions of the epimerization domains embedded in the enzymes. The obtained information is still utilized to predict epimerization domains in uncharacterized NRPSs. The second includes the biosynthetic enzymes of lantibiotics, which are ribosome-dependently supplied peptide antibiotics containing polycyclic thioether amino acids (lanthionines). A mechanism for the formation of the D-Ala moiety in lanthionine by two enzymes, dehydratases catalyzing the conversion of L-Ser into dehydroalanine and enzymes catalyzing nucleophilic attack of the thiol of cysteine into dehydroalanine, was clarified. Similarly, the formation of a D-Ala residue by reduction of the dehydroalanine residue was also reported. The last type of machinery includes radical-*S*-adenosylmethionine (rSAM)-dependent enzymes, which catalyze a variety of radical-mediated chemical transformations. In the biosynthesis of polytheonamide, a marine sponge-derived and ribosome-dependently supplied peptide composed of 48 amino acids, a rSAM enzyme (PoyD) is responsible for unidirectional epimerizations of multiple different amino acids in the precursor peptide. In this review, we briefly summarize the discovery and current mechanistic understanding of these peptide epimerization enzymes.

## UDP-*N*-Acetylmuramic Acid (UDP-MurNAc)-L-Ala-L-Glu Epimerase (MurL)

**FIGURE 1:**
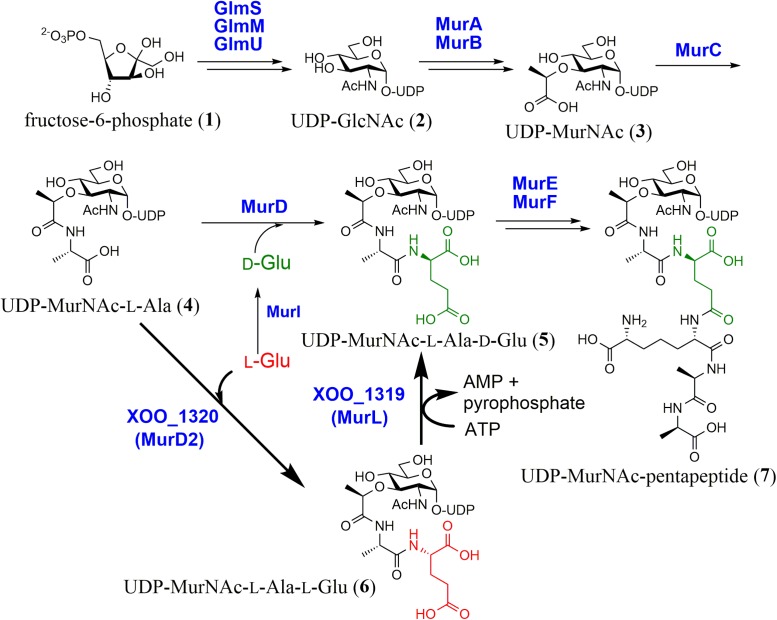
Biosynthesis of UDP-MurNAc-pentapeptide. A recently discovered pathway (shown with a bold arrow) employs a unique unidirectional peptide epimerase (XOO_1319, MurL).

Almost all eubacteria possess peptidoglycan to maintain cell integrity. Its biosynthetic pathway and mechanism have been well-studied. UDP-MurNAc-pentapeptide (**7**), a unit of peptidoglycan, is synthesized by nine enzymes (van Heijenoort, 1998; Gautam et al., 2011). In this pathway, UDP-MurNAc (**3**) is formed from fructose-6-phospate (**1**) via UDP-*N*-acetyl-glucosamine (UDP-GlcNAc, **2**) by five enzymes, GlmS, GlmM, GlmU, MurA, and MurB. Four structurally similar enzymes (MurC–F) then catalyze the successive addition of L-Ala, D-Glu, meso-diaminopimelate (DAP), and D-Ala-D-Ala to UDP-MurNAc (**3**) (**Figure 1**). In this process, D-Glu is supplied by either Glu racemases (typically MurI) or D-amino acid aminotransferases (Thorne et al., 1955; Doublet et al., 1993). To the best of our knowledge, the D-Glu formation mechanism is limited to these two enzymes in microorganisms.

We searched for an alternative biosynthetic pathway in the primary metabolisms of microorganisms and found the futalosine pathway for menaquinone biosynthesis (Hiratsuka et al., 2008; Dairi, 2012). In the same study, we found that *Xanthomonas oryzae* has no orthologs of the Glu racemase or the D-Glu aminotransferase, even though it possesses the other genes encoding enzymes for peptidoglycan biosynthesis. This suggested that *X. oryzae* biosynthesizes D-Glu via an alternative enzyme(s). We therefore investigated the mechanism (Feng et al., 2017).

We first carried out a shotgun cloning experiment with D-Glu auxotrophic *Escherichia coli* WM335 (Dougherty et al., 1993) and genomic DNA of *X. oryzae* MAFF311018 as donor DNA. We obtained DNA fragments that complemented the mutant, and further subcloning experiments revealed that two genes, XOO_1319 and 1320, which were annotated as a function unknown protein and MurD (UDP-MurNAc-L-Ala-D-Glu synthetase), respectively, were essential and neither XOO_1319 nor 1320 alone complemented the D-Glu auxotrophy.

We then investigated the functions of XOO_1319 and 1320 with recombinant enzymes. Because both genes were essential for complementation, we incubated both recombinant enzymes with UDP-MurNAc-L-Ala (**4**) and L-Glu in the presence of ATP and Mg^2+^, which are essential for the MurD reaction. By LC–MS analysis, we detected a product that had the same mass spectrum and elution time as UDP-MurNAc-L-Ala-D-Glu (**5**) formed by *E. coli* MurD with UDP-MurNAc-L-Ala (**4**) and D-Glu as substrates. Unexpectedly, no products were formed when D-Glu was used as the substrate instead of L-Glu even though XOO_1320 is similar to *E. coli* MurD. We therefore hypothesized that XOO_1320 and XOO_1319 were an UDP-MurNAc-L-Ala-L-Glu synthetase and an UDP-MurNAc-L-Ala-L-Glu epimerase, respectively. To confirm this hypothesis, recombinant XOO_1320 was incubated with UDP-MurNAc-L-Ala (**4**) and L-Glu. By LC–MS analysis, we detected a new product that had the same mass spectrum as UDP-MurNAc-L-Ala-D-Glu (**5**) but eluted at a different time. The chirality of the Glu moiety of the product (**6**) was confirmed to be L-Glu by a modified Marfey’s method. The results clearly showed that XOO_1320 was MurD utilizing L-Glu as the substrate (designated MurD2).

We next examined whether XOO_1319 had UDP-MurNAc-L-Ala-L-Glu epimerase activity. Recombinant XOO_1319 was incubated with UDP-MurNAc-L-Ala-L-Glu (**6**) in the presence of Mg^2+^ and ATP and the chirality of the terminal Glu of the product was examined. We confirmed that the terminal L-Glu of the substrate was converted into D-Glu, demonstrating that XOO_1319 was a novel type of glycopeptidyl-glutamate epimerase. We thus designated XOO_1319 as UDP-MurNAc-L-Ala-L-Glu epimerase, MurL. We also examined whether MurL catalyzed the D→L reaction with UDP-MurNAc-L-Ala-D-Glu (**5**) as the substrate. However, no epimerase activity was detected. Interestingly, MurL required ATP and Mg^2+^ for its activity and AMP was generated as a side product, suggesting that the substrate was activated by adenylation. However, the detailed reaction mechanism of this enzyme is not clear at this stage, because MurL lacks known conserved domains and cofactor-binding domains.

MurL orthologs are distributed among bacteria such as Gammaproteobacteria, actinobacteria, and Alphaproteobacteria. Orthologs with low similarity are also found in a plant pathogenic fungus, cyanobacteria, and an amoeba.

## L-Ala-D/L-Glu Epimerase

Gerlt et al. (2012) discovered “enolase superfamily” enzymes catalyzing at least 11 different reactions such as mandelate racemase, galactonate dehydratase, glucarate dehydratase, muconate-lactonizing enzymes, *N*-acylamino acid racemase, β-methylaspartate ammonia-lyase, and *o*-succinylbenzoate synthase (Babbitt et al., 1996). High resolution X-ray structures showed that the reactions catalyzed by these enzymes are initiated by a common reaction; Mg^2+^-assisted general base-catalyzed abstraction of the α-proton of a carboxylic acid and stabilization of an enolate anion intermediate. The fate of the intermediate is determined by the active site of each enzyme to produce the specific product.

In their study of “enolase superfamily” enzymes, Gerlt et al. discovered that *E. coli* and *Bacillus subtilis* possess orthologs of these enzymes, YcjG and YfkB, respectively (Schmidt et al., 2001). To predict the functions of these enzymes, they referred to genes located in the flanking regions of each gene. In the case of *ycjG*, two ORFs, an ortholog of endopeptidase catalyzing hydrolysis of the amide bond of D-Glu-meso-DAP in the peptidoglycan component and an ortholog of dipeptidyl peptidase, were identified. In *B. subtilis*, YkfC, which was homologous to the same endopeptidase, was located next to YktB. Moreover, there were no reports of a peptidase catalyzing the cleavage of L-Ala-D-Glu (**8**) and “enolase superfamily” enzymes catalyze reactions initiated by abstraction of the α-proton to form an enolate anion intermediate. Taking these observations together, they hypothesized that YcjG and YkfB were L-Ala-D/L-Glu epimerases, which convert L-Ala-D-Glu (**8**) into L-Ala-L-Glu (**9**) for degradation and recycling of peptidoglycan.

**FIGURE 2:**
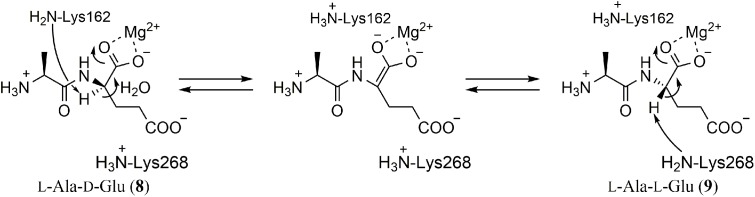
Reaction of L-Ala-D/L-Glu epimerases. A two-base reaction mechanism typical for enolase superfamily enzymes was postulated by X-ray crystallography and mutagenesis studies. The residue numbers are for YkfB.

Both recombinant enzymes were incubated with L-Ala-L-Glu (**9**) in D_2_O. After the reaction, epimerization of the L-Glu residue was confirmed by NMR and MS analysis. They also investigated substrate specificity with dipeptides composed of different amino acids. YcjG showed broad substrate specificity against dipeptides with L-Ala at the *N*-terminus but narrow specificity against dipeptides with L-Glu at the *C*-terminus. In contrast, YkfB had a narrow substrate specificity against both *N*- and *C*-terminal substrates. The kinetic parameters suggested that L-Ala-D/L-Glu was the intrinsic substrate for both enzymes (**Figure 2**).

The crystal structures of YcjG (apo form) and YkfB (apo form and the L-Ala-D/L-Glu complex) revealed that their overall structures were mostly identical to those of other enolase superfamily enzymes (Gulick et al., 2001; Klenchin et al., 2004). The structures comprised an *N*-terminal capping domain and a *C*-terminal (β/α)_7_β-barrel, and the active site was located in the barrel domain. As expected for the epimerization reaction, the Mg^2+^ ion formed a bidentate interaction with the α-carbonyl group of the Glu of the substrate and the α-carbon center to be epimerized was located between two conserved lysine residues (K162 and K268 in YkfB). Together with the fact that mutation of either of the two lysine residues reduced the epimerization activity, a two-base mechanism for the epimerization reaction was proposed (**Figure 2**). It is worth noting that these two lysine residues are also conserved among enolase superfamily enzymes and have been shown to be important for the abstraction of the α-proton of a carboxylic acid.

Orthologs of YkfB have been found in several microorganisms such as *Bacillus halodurans*, *Clostridium acetobutylicum*, *Clostridium difficile*, and *Thermotoga maritima*. Of these, the ortholog in *Thermotoga maritima* was characterized and shown to have high epimerase activity against L-Ala-D/L-Phe, L-Ala-D/L-Tyr and L-Ala-D/L-His (Kalyanaraman et al., 2008).

## Non-Ribosomal Peptide Synthetase (NRPS)

Non-ribosomal peptide synthetases (NRPSs) are modular-type large enzymes and a typical module consists of an adenylation (A) domain, a peptidyl carrier protein (PCP) domain and a condensation (C) domain (Crosa and Walsh, 2002; Finking and Marahiel, 2004; Marahiel, 2016). The A domain activates the carboxylic acid of an amino acid with ATP by adenylation and determines the amino acid to be selected and activated. Unlike ribosomes, NRPSs can utilize non-proteinogenic amino acids as building blocks. The C-domain catalyzes peptide bond formation between an upstream peptidyl PCP and a downstream amino acyl PCP. Besides the basic domains, additional domains catalyzing *N*-methylation, epimerization, and thiazoline/oxazoline formation, etc. have been reported.

**FIGURE 3:**
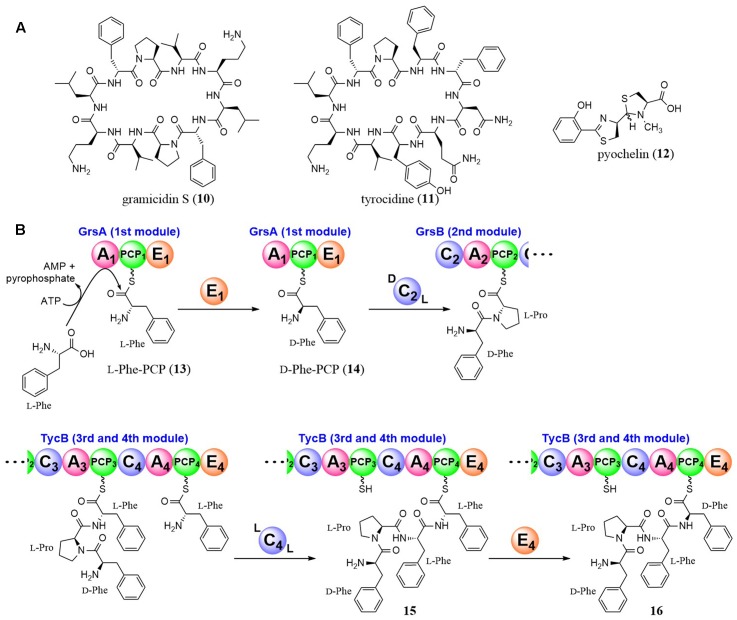
Structures of gramicidin *S*, tyrocidine, and pyochelin **(A)**, and epimerization reactions by NRPS E domains **(B)**.

The epimerization mechanism was investigated in the pioneering and excellent studies by Walsh et al. with gramicidin *S* (**10**) synthetase and tyrocidine (**11**) synthetase (**Figure 3**). They used the first module of gramicidin *S* synthetase for analysis, which is composed of three domains in the following order; an A domain for recognition and adenylation of L-Phe, a PCP domain for tethering L-Phe, and an E domain for epimerization of L-Phe (A-PCP-E domain) (Stachelhaus and Walsh, 2000; Luo and Walsh, 2001; Luo et al., 2001). By analysis of single-turnover catalysis using rapid chemical quench techniques, they showed that the reaction proceeded in the following order; disappearance of the substrate L-Phe, transient appearance and disappearance of L-Phe-AMP and formation of L-Phe-PCP (**13**) and D-Phe-PCP (**14**). They also showed that the C2 domain immediately downstream of the E1 domain is D-specific for the peptidyl donor and L-specific for the aminoacyl acceptor (^D^C_L_) (Clugston et al., 2003).

Tyrocidine (**11**) is a cyclic decapeptide produced by *Bacillus* strains and its amino acid sequence is D-Phe-L-Pro-L-Phe-D-Phe-L-Asn-L-Gln-L-Tyr-L-Val-L-Orn-L-Leu. Walsh et al. also investigated the epimerization mechanism of the D-Phe in the 1st and 4th residues (Luo et al., 2002). Although the mechanism of the 1st D-Phe formation and condensation with the 2nd amino acid (L-Pro) was the same as for gramicidin *S* synthetase as described above, the 4th D-Phe was revealed to be introduced after condensation. They demonstrated that the C4 domain in the TycB subunit of the tyrocidine synthetase utilized D-Phe-L-Pro-L-Phe as the peptidyl donor and L-Phe as the aminoacyl acceptor (^L^C_L_), showing that the two acyl units tethered to PCP domains were condensed before the epimerization (**Figure 3**). Then, the E4 domain acts on D-Phe-L-Pro-L-Phe-L-Phe-PCP (**15**) to yield a 1/1 mixture of D-Phe-L-Pro-L-Phe-D-Phe-PCP (**16**) and D-Phe-L-Pro-L-Phe-L-Phe-PCP (**15**). Subsequently, the downstream C5 domain selectively utilizes only D-Phe-L-Pro-L-Phe-D-Phe-PCP (**16**) as a substrate. These excellent studies brought us to the general conclusion that we can estimate the timing of epimerization by the relative positions of the E and C domains.

In the case of pyochelin (**12**), yersiniabactin, and micacocidin biosynthesis, all of which have a benzoyl moiety at the *N*-terminus and D-thiazolines derived from cyclodehydration of the *N*-acylcysteinyl precursor at the second position, the typical E domains are absent in their NRPSs. Walsh et al. examined the epimerization mechanism and showed that recombinant enzymes of pyochelin synthetase (PchE subunit) possesses the epimerase activity by *in vitro* experiments utilizing D_2_O and MS analysis (Patel et al., 2003). They confirmed that the PchE subunit did not epimerize a Cys-PCP intermediate, but epimerized *N*-benzoyl-Cys-PCP, an intermediate after the condensation between upstream benzoyl-PCP and Cys-PCP. Based on these results and mutagenesis studies, they suggested that a methyl transferase-like domain in the PchE subunit catalyzed the epimerization.

## Enzymes for Lantibiotic Biosynthesis

In 1994, Nes et al. cloned the structural gene of bacteriocin lactocin S and found that the codons corresponding to all three D-Ala residues in lactocin S were serine, suggesting that the D-Ala residues were biosynthesized by post-translational modifications of serine residues in the primary translated peptide product (Skaugen et al., 1994). After 10 years, van der Donk et al. solved this mystery by *in vitro* studies (Xie et al., 2004). They prepared recombinant LctA and LctM, which are 51-amino acid prepeptides and the probable multifunctional enzymes catalyzing dehydration and cyclization reactions, respectively, to form lanthionine in lacticin 481 (class II lanthipeptides) biosynthesis. When LctA and LctM were incubated in the presence of ATP and Mg^2+^, they detected a series of products with the reduced mass spectrum corresponding to the elimination of one to four water molecules from LctA, which resulted in the formation of dehydroalanine (Dha, **17**) and dehydrobutyrine (Dhb, **18**) from serine and threonine, respectively. They also detected ADP in the reaction mixture, suggesting that the substrate was activated by phosphorylation, which was later confirmed with a synthetic phosphorylated substrate (Chatterjee et al., 2005). Then, several experiments to support the formation of the lanthionine structure were performed because the mass of the dehydrated compound and the product with a lanthionine structure formed by adding cysteine residues to Dha/Dhb were the same. Finally, they confirmed the formation of the lanthionine structure using mass spectrometric studies together with biochemical analysis and bioassays.

Recently, van der Donk et al. reported that substrates (peptides) controlled the stereoselectivity of the enzyme-catalyzed Michael-type addition during the biosynthesis of class II lantipeptides, cytolysin and haloduracin (Tang et al., 2015). During the biosynthesis of lanthionine, the thiol of cysteine attacks dehydroalanine and usually results in the formation of DL-form lanthionine (D configuration at the α carbon originating from Ser and L configuration at the α carbon of Cys). However, in the case of cytolysin L, which has three lanthionine structures (A to C rings), they showed that the methyllanthionine (A ring) and lanthionine (B ring) residues have the LL configuration, whereas the C ring has the DL configuration, by heterologous co-expression of a precursor peptide with CylM catalyzing lanthionine formation in *E. coli*. They also showed that two consecutive dehydro amino acids in Dhb-Dhb-X-X-Cys motif (X represents amino acids other than Dha, Dhb, and Cys) in the precursor peptide was a key sequence for the formation of the LL configuration based on the sequence homology of the rings containing LL-methyllanthionine residues in cytolysin and haloduracin, suggesting that the sequence of the substrate peptide determines the stereoselectivity of lanthionine and methyllanthionine formation. Moreover, the two consecutive Dhb-Dhb sequences were shown to be important for the reaction by replacing the second Dhb of haloduracin with Ala. Thus, the stereochemistry of the Michael-type additions is unusually controlled by the substrate.

In the case of class I lantipeptide biosynthesis, two separate enzymes, LanB and LanC, which are a dehydratase and a cyclase, respectively, are employed for thioether linkage. Unlike LctM-type enzymes, LanB-type enzymes activate the hydroxyl group of Ser/Thr residues using glutamyl-tRNA (Garg et al., 2013; Ortega et al., 2015). Nair et al. succeeded in reconstituting the cyclization process of NisC in nisin biosynthesis *in vitro* (Li et al., 2006; Li and van der Donk, 2007). Moreover, its mechanism—an active site zinc ion bound by a cysteine-cysteine-histidine triad activates the thiol of cysteine for nucleophilic attack to Dha/Dhb—was also clarified by X-ray crystal structures.

Beside class I and II lantipeptide biosynthetic enzymes, class III lantipeptide biosynthetic enzymes composed of trifunctional domains, an *N*-terminal lyase domain, a central kinase domain, and a putative *C*-terminal cyclase domain, have been reported (Meindl et al., 2010). However, the cyclase domain lacks many of the conserved active-site residues found in class I and II enzymes. Recently, a class IV synthetase (LanL) containing an *N*-terminal lyase, a kinase domain, and a *C*-terminal cyclase domain similar to LanC was also reported (Goto et al., 2010, 2011). However, in both cases, the detailed reaction mechanisms of lanthionine structure (D-Ala) formation remain unknown.

**FIGURE 4:**
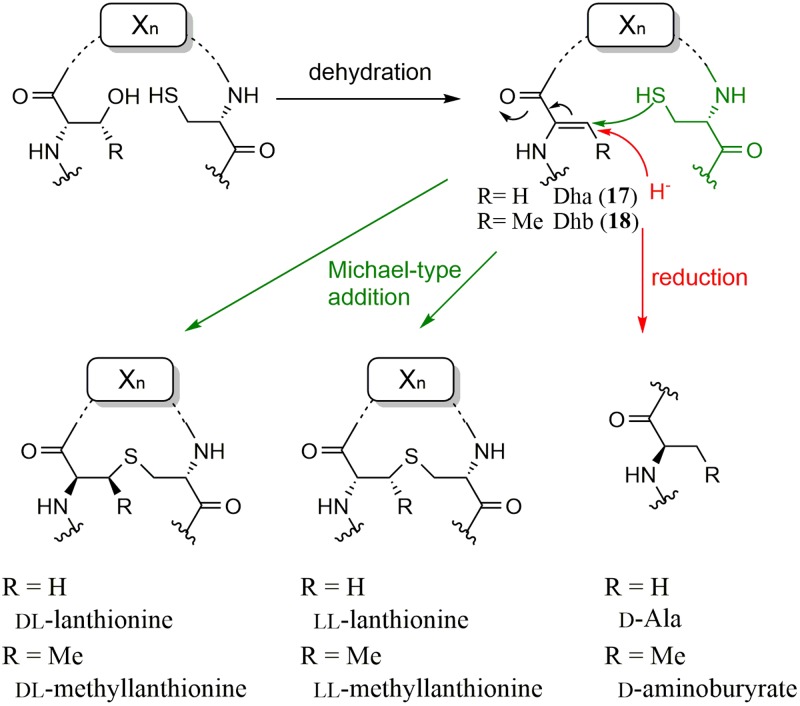
Formation mechanisms of D-amino acid residues discovered in the biosynthesis of lanthipeptides.

To date, three enzymes that catalyze D-Ala formation without the thioether linkage from Dha have been reported. Hill et al. identified an enzyme, LtnJ, which was responsible for the conversion of Dha to D-alanine in lacticin 3147 biosynthesis (Cotter et al., 2005). Vederas et al. recently identified CrnJ in the carnolysin biosynthetic gene cluster from *Carnobacterium maltaromaticum* (Lohans et al., 2014). By heterologous expression of the carnolysin cluster, they showed that D-alanine and D-aminobutyrate were formed from serine and threonine, respectively, by CrnJ. A NADPH-dependent Dha reductase catalyzing the conversion of Dha into D-Ala was reported by van der Donk et al. (Yang and van der Donk, 2015). A gene cluster for probable lantibiotic biosynthesis found in the cyanobacterium *Nostoc punctiforme* contains a gene encoding a prepeptide with several Ser/Thr-rich residues, which can be dehydrated into Dha and Dhb by a dehydratase also encoded in the cluster. However, there are no Cys residues in the prepeptide, which are usually used for thioether linkage. Therefore, they hypothesized that the cluster produced linear D-amino acid-containing peptides. Through an *in vitro* study with recombinant enzymes and MS analysis, they confirmed that NpnJ_A_, a dehydroalanine reductase, catalyzed the conversion of Dha into D-Ala. Recently, they also characterized a CrnJ-type reductase, BsjJ_B_ from *Bacillus cereus*, *in vitro* (Huo and van der Donk, 2016). The formation mechanisms of D-amino acid residues discovered in the biosynthesis of lanthipeptides are summarized in **Figure 4**.

## Radical *S*-Adenosylmethionine (rSAM) Enzymes

**FIGURE 5:**
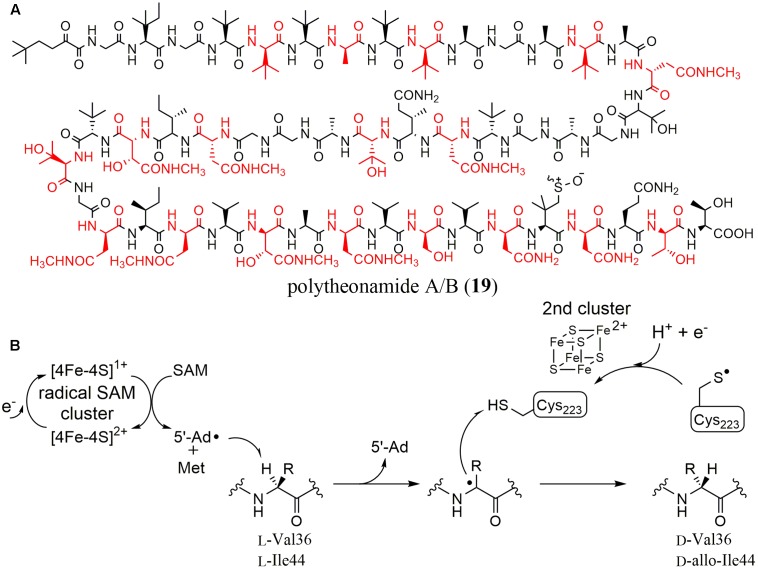
**(A)** Structures of polytheonamide A/B (differing in sulfoxide configuration). Amino acid residues with D-configuration are shown in red. **(B)** Reaction mechanism proposed for YydG.

Polytheonamides (**19**) are marine sponge-derived peptides composed of 48 amino acids (**Figure 5A**) (Hamada et al., 2005). Uniquely, they contain many modified amino acids such as eight *tert*-leucine, three β-hydroxyvaline, and six γ-*N*-methylasparagine residues. Moreover, 18 amino acids are D-form. Considering its complicated structure, polytheonamide was proposed to be biosynthesized by a NRPS. In 2012, however, Piel et al. succeeded in isolating its biosynthetic cluster (Freeman et al., 2012). They hypothesized that polytheonamide was biosynthesized by ribosomes and post-translationally modified (RiPPs) because polytheonamide is larger than other known peptides synthesized by NRPSs. They carried out PCR with primers that were designed using the amino acid sequences of a hypothetical precursor peptide composed of L-amino acids with the metagenome of the sponge as a template [later, a symbiotic microorganism was shown to produce polytheonamide (Wilson et al., 2014)]. They successfully amplified a specific DNA fragment encoding an ORF whose *C*-terminal amino acid sequence was perfectly consistent with the unprocessed polytheonamide precursor. In the flanking region, however, only 11 putative ORFs were found even though 48 post-translational modifications including 18 epimerizations are necessary for the maturation of polytheonamide. To examine the function of each of these genes, they expressed the individual ORFs in *E. coli*. No recombinant PoyA, which is the prepeptide of polytheonamide, was obtained but they found that the yield and solubility of PoyA was dramatically improved when PoyD, a rSAM enzyme, was co-expressed. Because the isolated PoyA had the same mass spectrum as calculated, they hypothesized that PoyD was an epimerase catalyzing the D-amino acid introduction, which results in no mass change. After acid hydrolysis and a derivatization to examine the chirality of the product, they showed that majority of the 18 amino acids of the precursor polytheonamide were epimerized, indicating PoyD is an epimerase and that epimerization from L- to D- configuration is unidirectional.

They then suggested that PoyD catalyzed all epimerizations in polytheonamide biosynthesis by co-expressing PoyA and full-length PoyA or truncated PoyA variants (Morinaka et al., 2014). Moreover, they showed that a leader region of PoyA, which is homologous to the α-subunit of nitrile hydratase proteins and composed of more than 100 amino acids, had an important function in the epimerization reaction. They utilized OspA and OspD, which are orthologs of PoyA and PoyD, respectively, from *Oscillatoria* sp. and confirmed that OspD introduced two epimerizations into OspA. They constructed OspA mutants by Ala replacement and co-expressed them in *E. coli* together with *ospD*. Although most of the mutants kept their epimerization activities, some mutants showed strongly reduced epimerization efficiencies.

Recently, another rSAM epimerase found in the *yyd* operon, YydG, was characterized *in vitro* (Benjdia et al., 2017). The *yyd* operon in *B. subtilis* was originally identified in the course of studying the cell wall stress response of firmicutes, and showed positive regulation upon cell-envelope stress signals, activating gene expression to maintain cell-wall integrity (Butcher et al., 2007). Because this operon contained genes encoding a putative precursor peptide (YydF), a rSAM enzyme (YydG), a protease (YydH) and ABC transporters (YydI and YydJ), a modified peptide was predicted as the biosynthetic product. However, the type of modification was not clear, because the corresponding metabolites were not identified and YydG did not have significant homology with any known rSAM enzymes. To characterize the function of YydG, Berteau et al. expressed the protein in *E. coli*. After incubating YydG with the full-length YydF peptide in the presence of DTT, they identified 5′-deoxyadenosine (5′-dA) and a modified YydF as the reaction products, which have identical molecular weights to the YydF peptide. Further analysis utilizing D_2_O and LC–MS/MS demonstrated that YydF epimerized the Cα of two residues (Val 36 and Ile 44) in the YydF peptide. They also showed that the purified and anaerobically reconstituted protein contained two [4Fe-4S] clusters.

They then further investigated the reaction mechanism of YydG. Considering that most rSAM enzymes initiate the reaction by generating a 5′-deoxyadenosyl radical (5′-dA), the reaction was envisioned to proceed by Cα hydrogen atom abstraction followed by the introduction of a hydrogen atom from the opposite side. Indeed, they showed that the 5′-deoxyadenosyl radical directly abstracted the Cα hydrogen atom using isotopically labeled YydF substrates. They also revealed that the hydrogen atom used to quench the Cα radical was provided by the thiol in the Cys 233 residue in YydG (**Figure 5B**).

## Concluding Remarks

In this review, we summarized the mechanisms of peptide epimerization found in prokaryotic microorganisms. To date, several enzymes that post-translationally introduce D-amino acids into peptides have been identified in eukaryotes (Ollivaux et al., 2014). The first such enzyme was discovered from the venom of the web spider. This epimerase exhibits homology to serine proteases, particularly in the region of the conserved catalytic triad, and acts on an amino acid residue near *C*-terminus of the peptide substrate. The second example is platypus-venom epimerase which showed similarity in its active site with aminopeptidases. Like aminopeptidases, this enzyme acts on an amino acid residue near the *N*-terminus of the substrate. In addition, a peptide epimerase that has weak homology *N*-terminal domain of the human IgG-Fc binding proteins was also identified in frog skin secretion. Although these enzymes are completely distinct proteins and have different active site residues, a two-base mechanism, a deprotonation/reprotonation of the α-carbon of the amino acid to be isomerized, is proposed for all cases. Conversely, several different machineries have been identified in microorganisms. Thus, prokaryotes and eukaryotes employ different strategies for the epimerization of peptides and microorganisms have evolved to have more divergent machineries.

## Author Contributions

YO and TD wrote the manuscript; YO prepared the figures. All authors approved the final manuscript.

## Conflict of Interest Statement

The authors declare that the research was conducted in the absence of any commercial or financial relationships that could be construed as a potential conflict of interest. The handling Editor declared a past collaboration and co-authorship with the authors.
